# Activation of PTEN by inhibition of TRPV4 suppresses colon cancer development

**DOI:** 10.1038/s41419-019-1700-4

**Published:** 2019-06-12

**Authors:** Xiaoyu Liu, Peng Zhang, Chuanming Xie, Kathy W. Y. Sham, Simon S. M. Ng, Yangchao Chen, Christopher H. K. Cheng

**Affiliations:** 1 Longgang E.N.T. hospital & Shenzhen Key Laboratory of E.N.T., Institute of E.N.T., Shenzhen, China; 2 School of Biomedical Sciences, The Chinese University of Hong Kong, Shatin, New Territories, Hong Kong, China; 3 Institute of Hepatobiliary Surgery, Southwest Hospital, The Third Military Medical University (Army Medical University), Chongqing, China; 4 Department of Surgery, Prince of Wales Hospital, The Chinese University of Hong Kong, Shatin, New Territories, Hong Kong, China

**Keywords:** Colon cancer, Oncogenes, Cell death, Cell growth

## Abstract

Transient receptor potential vanilloid type 4 (TRPV4) is a Ca^2+^-permeable cation channel that is known to be an osmosensor and thermosensor. Currently, limited evidence shows that TRPV4 plays opposite roles in either promoting or inhibiting cancer development in different cancer types. Furthermore, the precise biological functions and the underlying mechanisms of TRPV4 in carcinogenesis are still poorly understood. In this study, we demonstrated that TRPV4 is upregulated in colon cancer and associated with poor prognosis. Contrary to the reported cell death-promoting activity of TRPV4 in certain cancer cells, TRPV4 positively regulates cell survival in human colon cancer in vitro and in vivo. Inhibition of TRPV4 affects the cell cycle progression from the G1 to S phase through modulating the protein expression of D-type cyclins. Apoptosis and autophagy induced by TRPV4 silencing attenuate cell survival and potentiate the anticancer efficacy of chemotherapeutics against colon cancer cells. In addition, PTEN is activated by inhibition of TRPV4 as indicated by the dephosphorylation and increased nuclear localization. Knockdown of PTEN significantly abrogates TRPV4 silencing induced growth inhibition and recovers the capability of clonogenicity, as well as reduced apoptosis in colon cancer cells. Thus, PTEN regulates the antigrowth effects induced by TRPV4 inhibition through both phosphatase-dependent and independent mechanisms. In conclusion, inhibition of TRPV4 suppresses colon cancer development via activation of PTEN pathway. This finding suggests that downregulation of TPRV4 expression or activity would conceivably constitute a novel approach for the treatment of human colon cancer.

## Introduction

Colorectal cancer is the third most common malignant type of cancer and the fourth leading cause of cancer-related deaths worldwide^1^. Due to the complex and heterogeneous pathogenic mechanisms that lead to colon cancer, and despite improving surgical and adjuvant treatment approaches, colon cancer is still a significant public health challenge.

Ca^2+^ homeostasis controls various cellular and physiological processes, including tumorigenesis^2^. Abnormal expression and activation of specific Ca^2+^ channels have been widely reported in several types of cancer^3^. Emerging evidence demonstrated that transient receptor potential (TRP) cation channels have multiple functions in cancer, ranging from controlling Ca^2+^ homeostasis to the regulation of tumorigenic and metastatic events^4^. TRP vanilloid type 4 (TRPV4) is a Ca^2+^-permeable nonselective cation channel that belongs to the vanilloid subfamily of TRP channels. TRPV4 is an integrator of diverse stimuli including moderate heat, mechanical stimulation, shear stress, endogenous chemicals and other chemical ligands^5^. Thus, TRPV4 is a major player in not only maintaining vascular tone and pain perception but also in cardiac, respiratory, urinary, skeletal, nervous, and digestive systems, as well as in tumorigenesis^6,7^. In previous studies, it was demonstrated that TRPV4 was highly expressed in tumor-derived endothelial cells and the absence of TRPV4 induced increased vascular density and enhanced tumor growth in lung cancer^8^. TRPV4 was involved in Ca^2+^-induced cell proliferation, migration, and invasion in gastric cancer^9^. However, TRPV4 was downregulated in keratinocytes derived from human nonmelanoma skin cancer^10^. Moreover, elevated TRPV4 expression was predominately found in a specific subset of basal molecular breast cancer and that TRPV4 activation led to reduced tumor growth^11^. But, in breast tumor-derived endothelial cells, TRPV4 activation by arachidonic acid promoted cell growth and migration^12^. Thus, in different types of cancer TRPV4 may be either oncogenic or tumor suppressive. Thus the underlying mechanisms by which TRPV4 regulates cancer cell growth remain to be elucidated. Furthermore, the role of TRPV4 in colon cancer has not yet been identified. This study represents the first study on TRPV4 in colon cancer and we aimed at elucidating the functional significance and molecular mechanism of TRPV4. Our results indicated that TRPV4 was upregulated in colon cancer and associated with poor prognosis. Furthermore, inhibition of TRPV4 suppressed the development of human colon cancer in vitro and in vivo through activation of PTEN signaling.

## Results

### TRPV4 is upregulated in primary human colon cancer

**Fig. 1:**
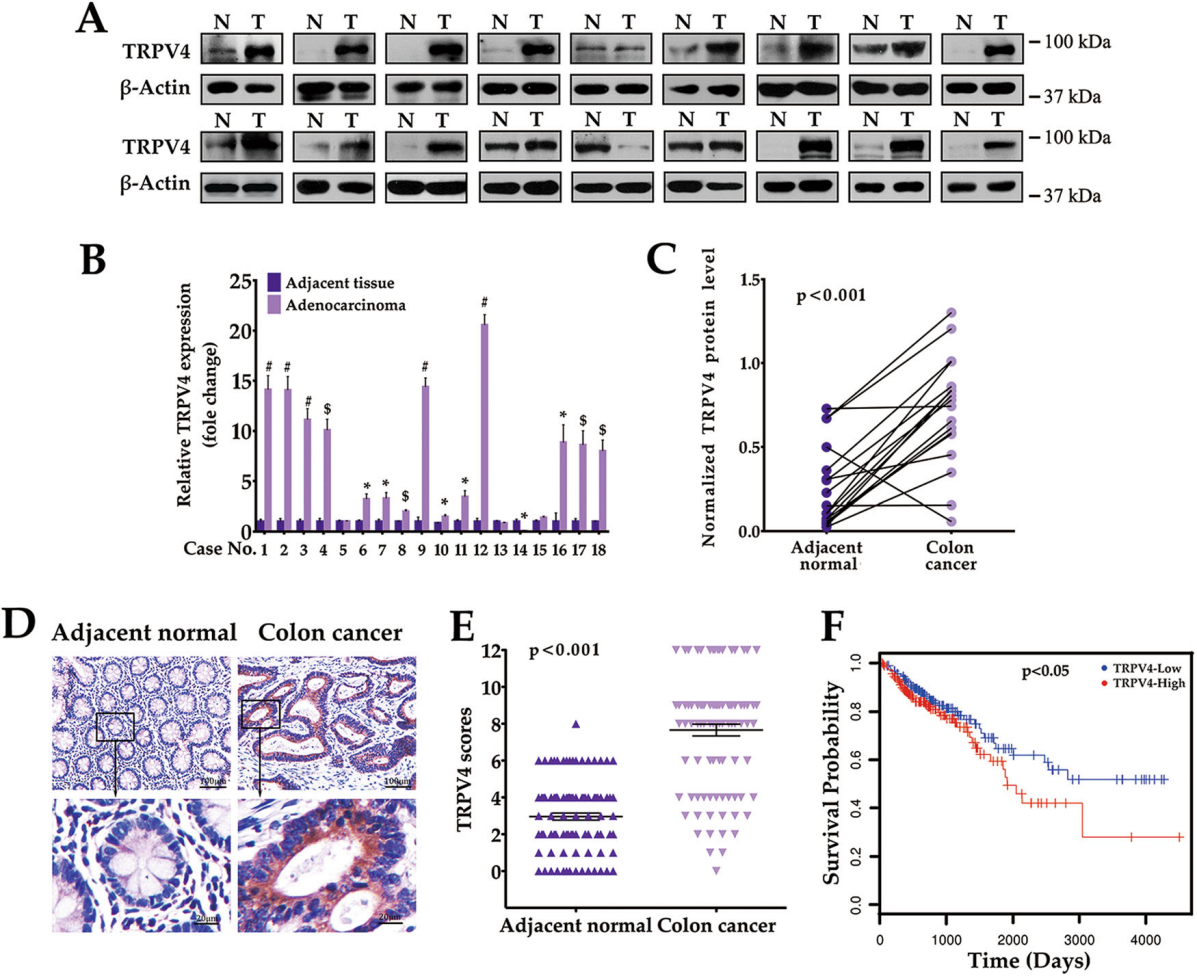
TRPV4 expression is elevated in colon cancer patients. **a** Representative western blot images of total lysates extracted from human colon cancer and matched adjacent normal tissues (normalized to β-actin). **b**, **c** Quantitative immunoblot analysis of TRPV4 protein level in colon cancer tissues and matched normal control from 18 subjects. **d** Representative images of TRPV4 protein expression in colon cancer tissue and matched adjacent normal tissue by immunohistochemistry. **e** TRPV4 expression scores were displayed in scatter plot. **f** Kaplan–Meier plots of colon cancer patients with high and low TRPV4 expression. All quantitative data shown represent the means ± SEM of at least three independent experiments. ^*^P < 0.05, ^$^*P* < 0.01 and ^#^*P* < 0.001, versus the adjacent normal group (for **b**)

To investigate the potential clinical role of TRPV4 in colon cancer, we first examined TRPV4 protein expression in cancer as well as in matched adjacent normal tissues from 18 human subjects (Fig. 1a). Analysis of band densities revealed that in 78% (14/18) of colon cancer cases, TRPV4 expression was approximately eightfold higher when compared to normal tissues (Fig. 1b, c). Next, we assessed TRPV4 expression by immunohistochemistry (IHC) using a tissue array consisting of 100 pairs of human colon cancer and matched nontumor colon tissues (Fig. 1d, e). Our data showed that in 86% (86/100) of patients, TRPV4 expression levels in colon cancer were higher when compared to adjacent normal tissues. We further evaluated the prognostic value of TRPV4 in the Cancer Genome Atlas database, in which TRPV4-high patients were found to have reduced overall survival time when compared with TRPV4-low patients^13^ (Fig. 1f). Together, these data suggested an aberrant upregulation of TRPV4 in colon cancer.

### Functional TRPV4 channels are present in colon cancer cells

**Fig. 2:**
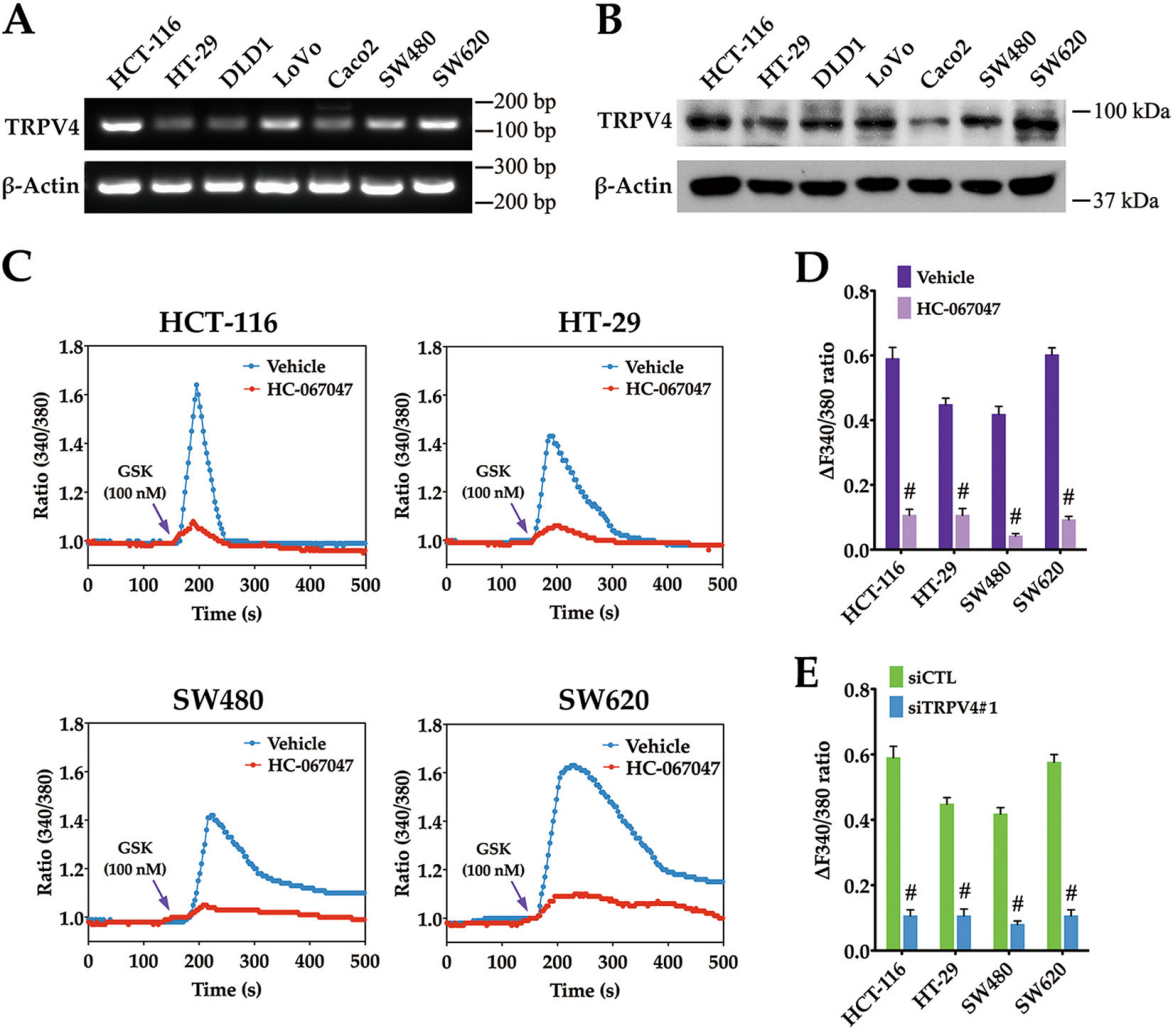
Functional TRPV4 channels are present in colon cancer cells. RT-PCR analysis of TRPV4 mRNA expression (**a**) and western blot analysis of TRPV4 protein expression (**b**) in indicated colon cancer cells. β-actin was used as the loading control. **c**, **d** Representative images and summary data from intracellular Ca^2+^ measurement in response to 100 nM GSK1016790A (agonist, arrowhead) in HCT-116, HT-29, SW480 and SW620 cells that were pretreated with vehicle (0.1% DMSO) or HC-067047 (4 μΜ). **e** Summary data from intracellular Ca^2+^ measurement in response to 100 nM GSK1016790A in HCT-116, HT-29, SW480 and SW620 cells that were transfected with control siRNA (siCTL) or TRPV4 siRNA (siTRPV4#1). All quantitative data shown represent the means ± SEM of at least three independent experiments. ^#^*P* < 0.001, versus vehicle treatment only (**d**) or the siCTL group (**e**)

To investigate the pathophysiologic role of TRPV4 in colon cancer, we verified the expression and function of TRPV4 channels in colon cancer cell lines. First, TRPV4 mRNA and protein expression have been evaluated in seven colon cancer cell lines (Fig. 2a, b). GSK1016790A, a selective TRPV4 activator^14^, was used to study the functional effect of TRPV4 activation. Fura-2 imaging of Ca^2+^ activity showed that GSK1016790A produced rapid and sustained elevation of intracellular Ca^2+^ level in colon cancer cells. These elevations were attenuated by a specific TRPV4 channel blocker HC-067047^15^ or by TRPV4 siRNA (Fig. 2c–e). Together, these results suggested that Ca^2+^-permeable TRPV4 channels are present in colon cancer cells.

### Inhibition of TRPV4 activity or expression suppresses colon cancer cell growth

**Fig. 3:**
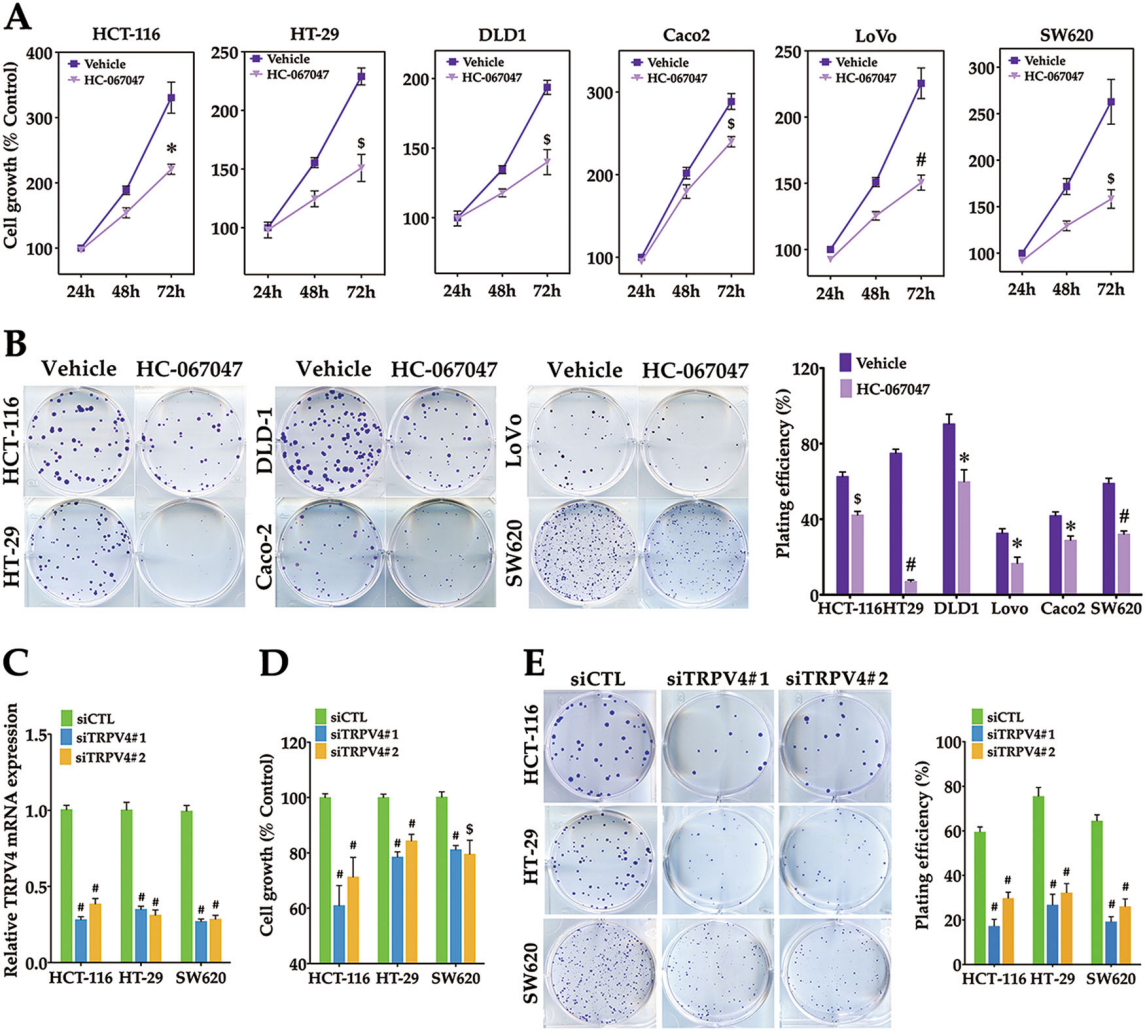
Inhibition of TRPV4 activity or expression suppresses colon cancer cell growth. **a** The effect of HC-067047 treatment on cell viability. The indicated colon cancer cells were treated with vehicle (0.1% DMSO) or HC-067047 (4 μΜ) and then assessed by MTT assay. **b** The effect of HC-067047 treatment on colony formation. The indicated colon cancer cells were seeded into six-well plates, then treated with vehicle (0.1% DMSO) or HC-067047 (4 μΜ), incubated at 37 °C for 12–14d, stained with crystal violet (0.5% w/v) and imaged. Colonies with 50 or more cells were counted. **c** Summary data from real-time PCR demonstrating the knockdown efficiency of TRPV4 siRNA in HCT-116, HT-29 and SW620 cells. Cells were transfected with control siRNA (siCTL), TRPV4 siRNA#1(siTRPV4#1) or TRPV4 siRNA#2 (siTRPV4#2) for 24 h. **d** The effect of TRPV4 knockdown on cell viability. HCT-116, HT-29 or SW620 cells were transfected as in (**c**), and then assessed by the MTT assay for 72 h. **e** The effect of TRPV4 knockdown on colony formation. HCT-116, HT-29 or SW620 cells were transfected as in (**c**). After 48 h transfection, cells were seeded into six-well plates, incubated and stained as in (**b**). All quantitative data shown represent the means ± SEM of at least three independent experiments. ^*^P < 0.05, ^$^*P* < 0.01 and ^#^*P* < 0.001, versus vehicle treatment only (**a**, **b**) or the siCTL group (**c**, **d**, **e**)

Ca^2+^ is essential for cell growth. We next investigated whether TRPV4 plays a role in colon cancer cell growth. First, we determined the effect of HC-067047 on cell growth of six colon cancer cell lines. After treatment of these cell lines with HC-067047, the growth capacity and the clonogenesis ability were inhibited (Fig. 3a, b). To confirm these findings, two different siRNAs for TRPV4 were transfected into HCT-116, HT-29, and SW620 cells. Real-time PCR analysis revealed that TRPV4 siRNAs reduced mRNA expression level by 60–70% (Fig. 3c). In addition, cell growth was substantially reduced when TRPV4 was downregulated by these siRNAs (Fig. 3d). In line with these findings, the number of colonies formed was reduced in TRPV4-depleted HCT-116, HT-29, and SW620 cells (Fig. 3e). Taken together, these results demonstrated that blocking the activity or expression of TRPV4 inhibited colon cancer cell growth.

### TRPV4 channels are essential for G1/S phase transition and the translation of D-type cyclins in colon cancer cells

**Fig. 4:**
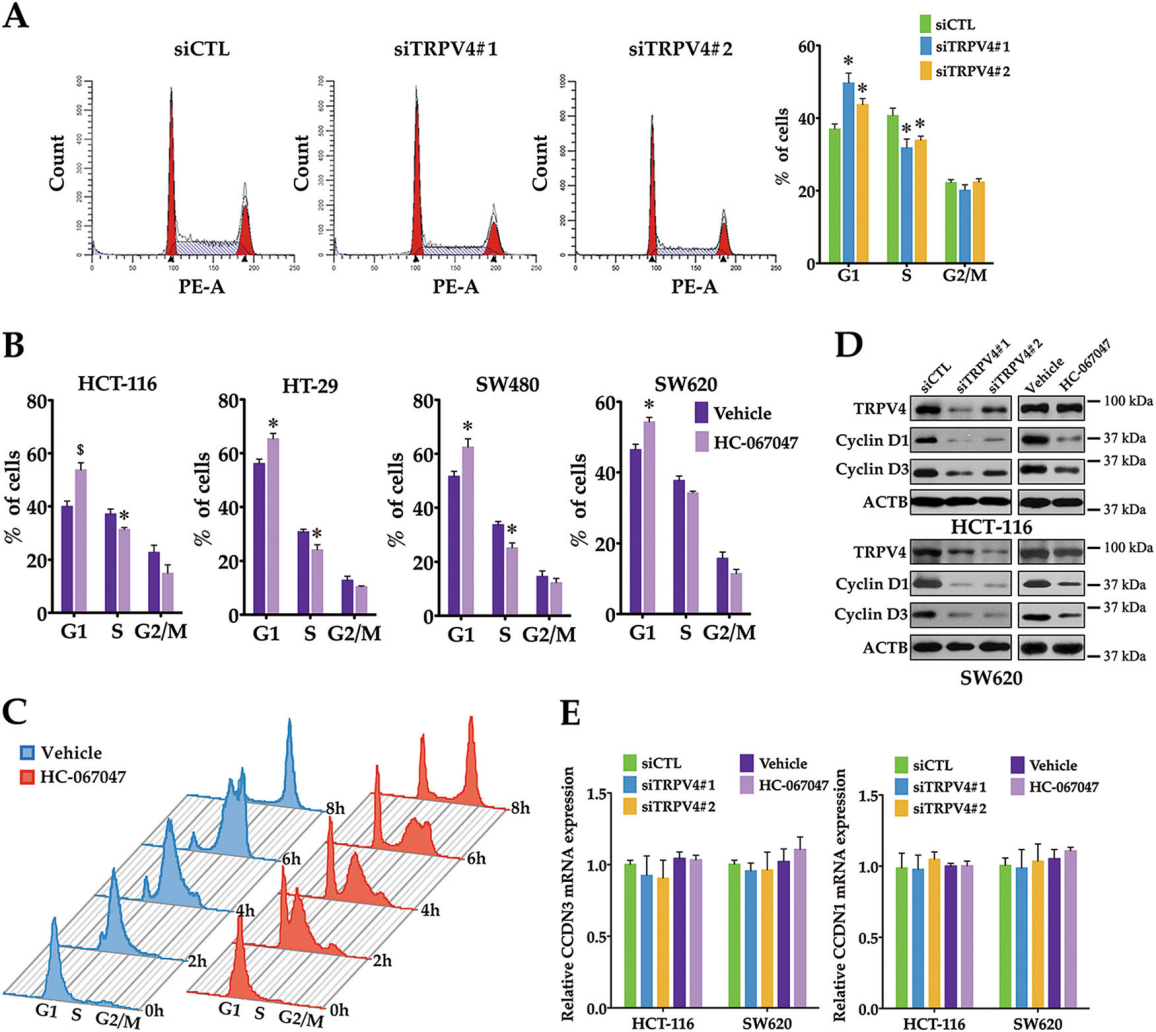
Inhibition of TRPV4 activity or expression arrests colon cancer cell on G1/S phase. **a** The effect of TRPV4 knockdown on cell cycle distribution. HCT-116 cells were transfected with control siRNA (siCTL), TRPV4 siRNA#1(siTRPV4#1) or TRPV4 siRNA#2 (siTRPV4#2) for 48 h, and then cell cycle distribution was determined by PI staining. **b** The effect of HC-067047 treatment on cell cycle distribution. HCT-116, HT-29, SW480 or SW620 cells were treated with vehicle (0.1% DMSO) or HC-067047 (4 μΜ) for 48 h, and then cell cycle distribution was determined by PI staining. **c** HCT-116 cells were synchronized to the G1/S boundary by double-thymidine block and then released into fresh medium in the presence of vehicle (0.1% DMSO) or HC-067047 (4 μΜ) for different time intervals. And cell cycle distribution was determined by PI staining. **d** TRPV4 knockdown or HC-067047 downregulates the protein levels of cyclin D1 and cyclin D3 in colon cancer cells. HCT-116 and SW620 cells were transfected as in (**a**) or treated as in (**b**). cyclin D1, cyclin D3, and β-actin (ACTB) were analyzed by western blot. **e** TRPV4 knockdown or HC-067047 does not change the mRNA levels of cyclin D1 and cyclin D3. HCT-116 and SW620 cells were transfected as in (**a**) or treated as in (**b**). The mRNA levels of cyclin D1 and cyclin D3 were analyzed by real-time PCR. All quantitative data shown represent the means ± SEM of at least three independent experiments. ^*^P < 0.05 and ^$^*P* < 0.01 versus the siCTL group (**a**) or vehicle treatment only (**b**)

To characterize the oncogenic mechanism of TRPV4 in colon cancer cell growth, we investigated the function of TRPV4 in cell cycle progression by flow cytometry. As shown in Fig. 4a, we demonstrated that downregulation of TRPV4 in HCT-116 cells increased the proportion of cells in the G1 phase, and decreased the proportion of cells in the S phase when compared with control siRNA-transfected cells. Consequently, inhibiting TRPV4 activity by treatment with HC-067047 arrested the cell cycle at the G1 transition in HCT-116, HT-29, SW480, and SW620 cells (Fig. 4b). To confirm the function of TRPV4 in G1/S phase transition, HCT-116 cells were synchronized at the G1/S boundary by double-thymidine treatment, then released in the presence of vehicle or HC-067047 for 2, 4, 6, and 8 h, respectively. As shown in Fig. 4c, the percentage of cells entering the S phase decreased in the HC-067047 treated group when compared with the control group. These results suggested that TRPV4 was critical for G1 to S transition in colon cancer cells. Moreover, western blot analysis showed that protein expression of cyclin D1 and D3, both master G1/S checkpoint regulators, were decreased in TRPV4 knockdown or HC-067047 treated HCT-116 or SW620 cells when compared with the control group (Fig. 4d). To determine whether the reduction in protein level of cyclin D1 and cyclin D3 was due to a reduction of mRNA levels, real-time PCR was performed. The results showed that cyclin D1 and D3 mRNA levels were not affected by blocking the expression or activity of TRPV4 (Fig. 4e). These findings suggested that the primary effect of inhibiting TRPV4 on cyclin D1 and D3 expression was probably exerted at the post-transcriptional level.

### Silencing of TRPV4 induces apoptosis in colon cancer cells

**Fig. 5:**
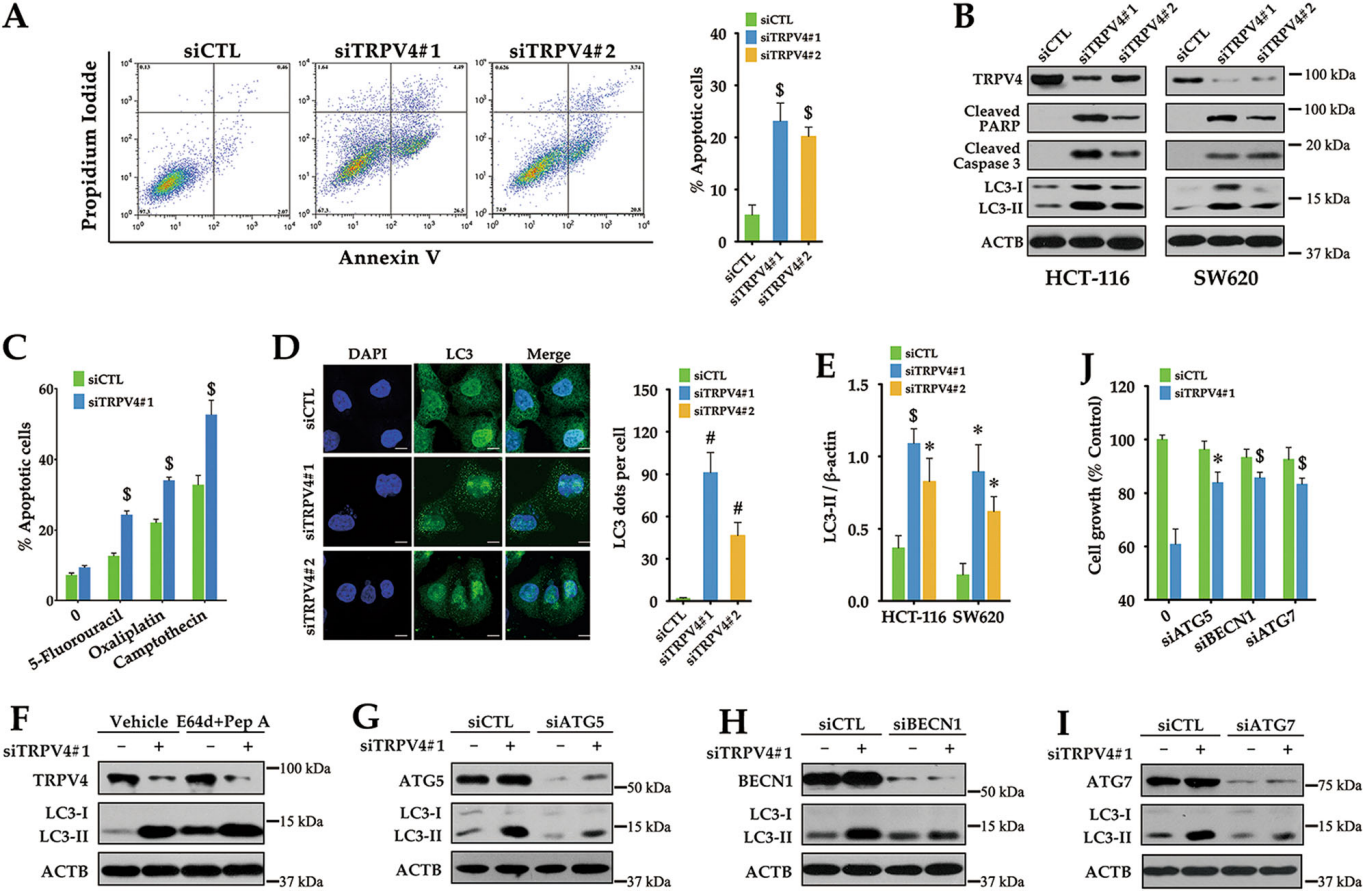
Silencing of TRPV4 induces apoptosis and autophagy in colon cancer cells. **a** The effect of TRPV4 knockdown on apoptosis. HCT-116 cells were transfected with control siRNA (siCTL), TRPV4 siRNA#1(siTRPV4#1) or TRPV4 siRNA#2 (siTRPV4#2) for 72 h, and then apoptosis was determined by Annexin V/PI staining. **b** Representative western blot analysis of cleaved PARP, cleaved Caspase 3, LC3 and β-actin (ACTB) in TRPV4-silenced HCT-116 or SW620 cells. **c** The effect of TRPV4 knockdown on anticancer drug-induced apoptosis. HCT-116 cells were transfected with control siRNA (siCTL) or TRPV4 siRNA#1(siTRPV4#1) for 24 h, and then treated with vehicle (0.1% DMSO), 5-fluorouracil (384 μM), oxaliplatin (50 μM) or camptothecin (0.5 μM) for 24 h. Apoptosis was determined by Annexin V/PI staining. **d** Representative immunofluorescent images showing redistribution of the autophagic marker LC3 dots in TRPV4 knockdown colon cancer cells captured on a confocal microscope. Scale bar: 10 μm. The average number of LC3 dots per cell was counted in more than 5 fields with at least 100 cells for each group. (**e**) Densitometric analysis normalized to β-actin demonstrating the effect of TRPV4 silencing on LC3-II levels. **f** Representative western blot analysis demonstrating the effect of lysosomal protease inhibitors E64d plus pepstatin A (Pep A) on TRPV4 silencing induced LC3-II accumulation. HCT-116 cells were transfected with control siRNA (siCTL) or TRPV4 siRNA#1(siTRPV4#1). At 3 h after transfection, cells were treated with 10 μg/ml E64d and Pep A for 69 h. (**g**, **h**, **i**) Representative western blot analysis demonstrating the effects of ATG5 siRNA (**g**), BECN1 siRNA (**h**) and ATG7 siRNA (**i**) on LC3-II levels induced by TRPV4 silencing. HCT-116 cells were transfected with control siRNA (siCTL), TRPV4 siRNA#1 (siTRPV4#1), ATG5 siRNA (siATG5), BECN1 siRNA (siBECN1), ATG7 siRNA (siATG7), siTRPV4#1 plus siATG5, siTRPV4#1 plus siBECN1 or siTRPV4 plus siATG7 for 72 h. **j** The effects of ATG5 siRNA, BECN1 siRNA, and ATG7 siRNA on the decrease of cell viability induced by TRPV4 silencing. HCT-116 cells were transfected as in (**g**, **h**, **i**) for 72 h. Cell viability was assessed by the MTT assay. All quantitative data shown represent the means ± SEM of at least three independent experiments. ^*^P < 0.05, ^$^*P* < 0.01, and ^#^*P* < 0.001, versus the siCTL group (**a**, **c**, **d**, **e**) or versus the siTRPV4#1 group (**j**)

Concomitant with cell cycle arrest, the growth-inhibitory effect of TRPV4 knockdown may also be related to the induction of cell death. Annexin V/PI staining was performed to determine the effect of TRPV4 on apoptosis. Our data showed an increased number of apoptotic cells in TRPV4-silenced HCT-116 cells (Fig. 5a). Moreover, silencing of TRPV4 enhanced protein levels of cleaved caspase-3, which is responsible for apoptosis execution, and PARP, which is the caspase-3 substrate during apoptosis (Fig. 5b). In addition, silencing of TRPV4 potentiated the anticancer efficiency of 5-fluorouracil, oxaliplatin, and camptothecin against colon cancer cells (Fig. 5c). Taken together, our results indicated that inhibition of TRPV4 expression contributed to apoptosis in colon cancer cells.

### Silencing of TRPV4 induces autophagy in colon cancer cells

Autophagy represents another type of cell death. We have investigated whether autophagy also participated in TRPV4 silencing-induced cell death. As shown in Fig. 5b, e, TRPV4 silencing increased the amount of LC3-II in both HCT-116 and SW620 cells. These findings were further substantiated by the accumulation of LC3 puncta in the cytoplasm of HCT-116 cells (Fig. 5d). Furthermore, E64d plus pepstatin A, the protease inhibitors, further increased the LC3-II level in TRPV4-silenced cells, suggesting that LC3-II accumulation in TRPV4-silenced cells was attributed to the promotion of autophagy but not to the impairment of autophagic degradation (Fig. 5f). ATG5, BECN1, and ATG7 are autophagy-related genes which take part in the process of autophagy. In previous studies, it was shown that autophagy can be induced through ATG5-, BECN1- or ATG7-dependent or independent pathways. To determine whether ATG5, BECN1, or ATG7 are required for autophagy in response to TRPV4 silencing, we used the siRNA approach to silence ATG5, BECN1, or ATG7 in HCT-116 cells. The data showed that knockdown of ATG5, BECN1, or ATG7 attenuated the accumulation of LC3-II in TRPV4-silenced cells (Fig. 5g–i). In cancer cells, autophagy is associated with either cell survival or cell death^16^. In order to identify the role of TRPV4 silencing induced autophagy, we silenced TRPV4 and autophagy-related genes at the same time, then measured the cell viability. As shown in Fig. 5j, knockdown of autophagy-related genes plus TRPV4 increased cell viability, compared to TRPV4 silencing group. Thus, TRPV4 silencing-induced autophagy promotes colon cancer cell death.

### Inhibition of TRPV4 activity or expression suppresses the development of xenografted colon cancer cells

**Fig. 6:**
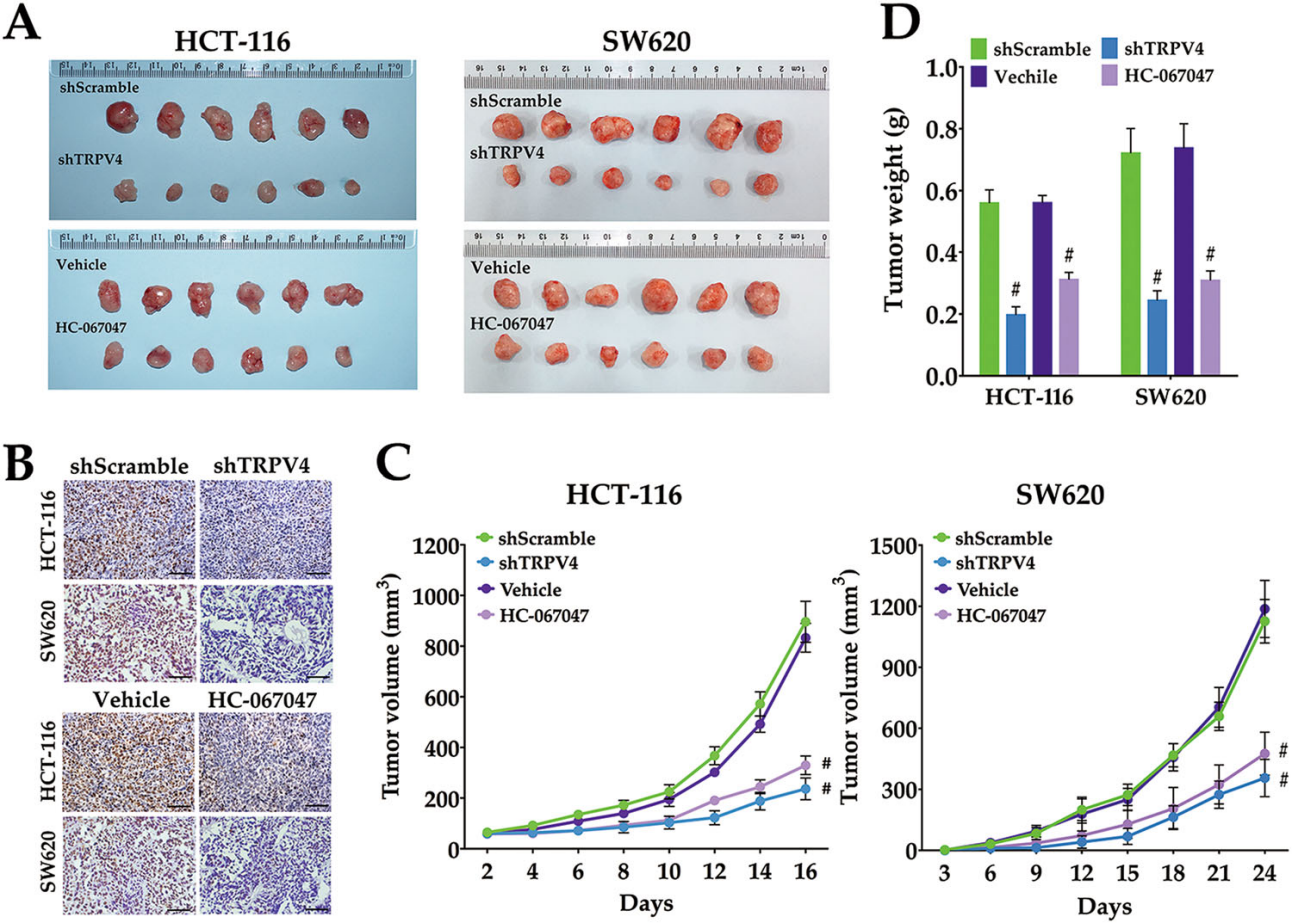
Inhibition of TRPV4 expression or activity suppresses colon cancer cell growth in vivo. **a** The effect of TRPV4 knockdown or HC-067047 on a xenograft model in vivo. The upper panel represents the xenograft tumors of mice (*n* = 6) that were injected with HCT-116 or SW620 cells stably transfected with scrambled-shRNA (shScramble) or TRPV4-shRNA (shTRPV4). The lower panel represents xenograft tumors of mice (*n* = 6) that were injected with HCT-116 or SW620 cells then treated with vehicle (0.1% DMSO) or HC-067047 (4 μΜ) every 2 days. **b** Representative images of IHC staining of Ki-67 in xenograft tumor tissue. **c** The tumor growth curve of the xenograft model. The tumor volumes were measured once every 2 days (HCT-116) or 3 days (SW620). **d** The average tumor weight (*n* = 6) was measured after the mice were harvested. All quantitative data shown represent the means ± SEM of six mice. ^#^*P* < 0.001, versus the shScramble group or versus vehicle group

To provide direct evidence that TRPV4 channels are responsible for the tumorigenic ability of colon cancer cells, we subcutaneously injected HCT-116 or SW620 cells that were infected with shScramble or shTRPV4 into the right flank of nude mice. We found that treatment with TRPV4 shRNA resulted in a significant reduction in tumor volume and weight compared with the shScramble group (Fig. 6a, c, d). Moreover, tumors from nude mice injected with shTRPV4-transfected cells displayed markedly decreased proliferative activity when compared with the shScramble-transfected group as determined by Ki-67 immunostaining (Fig. 6b). Similarly, blocking the activity of TRPV4 by HC-067047 also attenuated tumorigenesis in vivo (Fig. 6a–d). Data from the in vivo model provided evidence that inhibition of TRPV4 expression or activity suppressed the development of xenografted HCT-116 and SW620 cells.

### Silencing of TRPV4 inhibits cyclin D translation by preventing AKT-mediated inactivation of mTOR

**Fig. 7:**
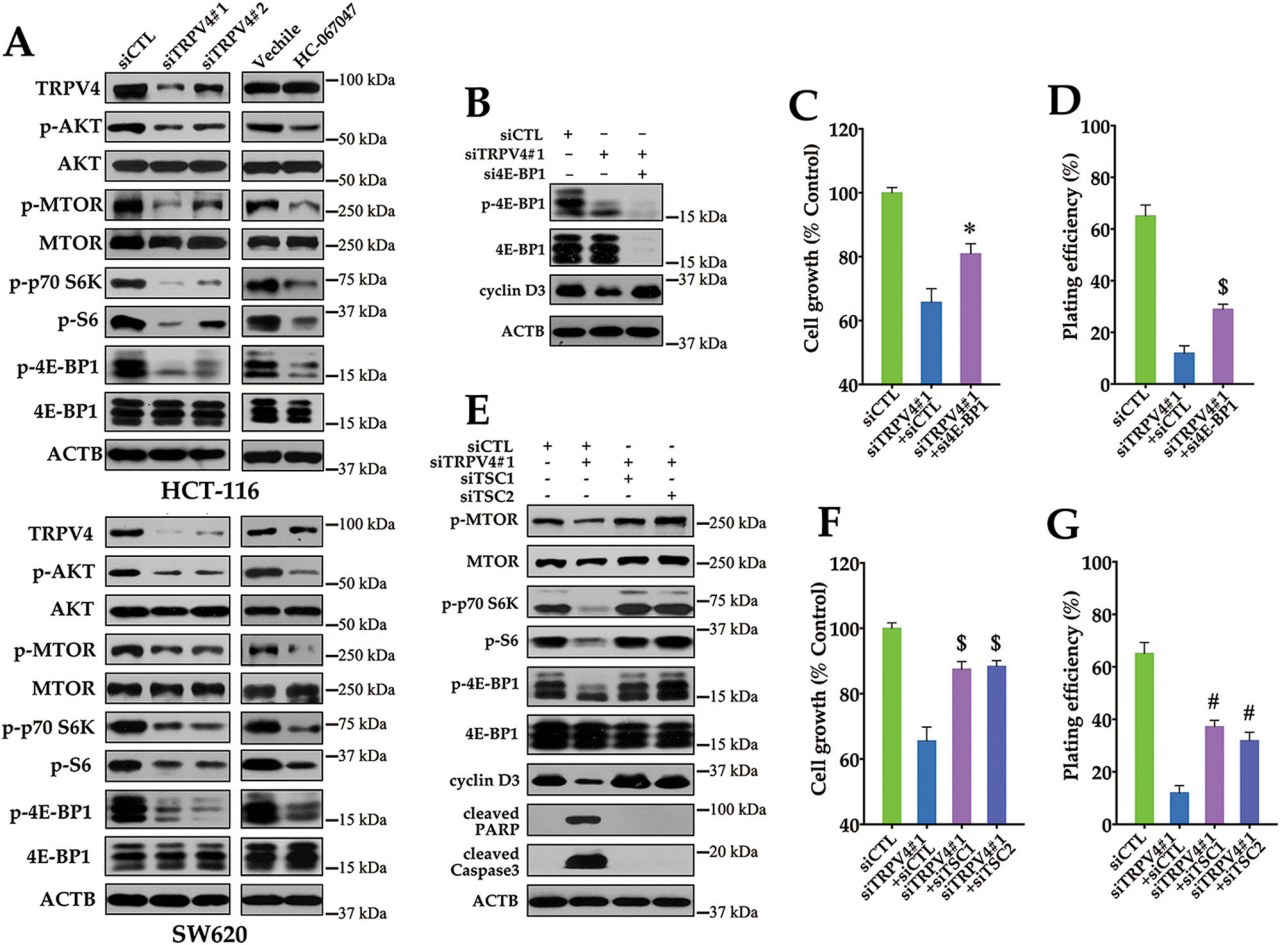
The AKT-mTOR pathway is required for cell growth inhibition induced by TRPV4 silencing. **a** TRPV4 knockdown or HC-067047 inhibits AKT-mTOR signaling in colon cancer cells. HCT-116 or SW620 cells were transfected with control siRNA (siCTL), TRPV4 siRNA#1(siTRPV4#1) or TRPV4 siRNA#2 (siTRPV4#2) for 72 h, or treated with vehicle (0.1% DMSO) or HC-067047 (4 μΜ). The protein levels of TRPV4, phospho-AKT (Ser473; p-AKT), AKT, phospho-mTOR (Ser2448; p-mTOR), mTOR, phosphor-p70 S6 Kinase (Thr389; p-p70S6K), phosphor-S6 Ribosomal Protein (Ser235/236; p-S6), phospho-4E-BP1 (Thr37/46; p-4E-BP1); 4E-BP1, and ACTB were analyzed by western bolt. **b** The effect of 4E-BP1 siRNA (si4E-BP1) on decrease of cyclin D3 expression induced by TRPV4 silencing. HCT-116 cells were transfected with siCTL, siTRPV4#1 plus siCTL, or siTRPV4#1 plus si4B-BP1 for 72 h. **c** The effect of 4E-BP1 siRNA on the decrease of cell viability induced by TRPV4 silencing. Cell viability was analyzed by MTT assay. **d** The effect of 4E-BP1 siRNA on the decrease of colony formation induced by TRPV4 silencing. **e** The effects of TSC1 siRNA (siTSC1) and TSC2 siRNA (siTSC2) on the inhibition of mTOR signaling, the decrease of cyclin D3 expression or the increase of apoptosis marker cleaved PARP and Caspase3 expression induced by TRPV4 silencing. HCT-116 cells were transfected with siCTL, siTRPV4#1 plus siCTL, siTRPV4#1 plus siTSC1 or siTRPV4#1 plus siTSC2 for 72 h. **f** The effects of TSC1 siRNA and TSC2 siRNA on the decrease of cell viability induced by TRPV4 silencing. **g** The effects of TSC1 siRNA and TSC2 siRNA on the decrease of colony formation induced by TRPV4 silencing. All quantitative data shown represent the means ± SEM of at least three independent experiments. ^*^P < 0.05, ^$^*P* < 0.01 and ^#^*P* < 0.001, versus the siTRPV4#1 plus siCTL group

Our results indicated that TRPV4 regulated cyclin D1 and D3 expression via a post-transcriptional mechanism. mTOR regulates protein synthesis through activation of p70S6K and inactivation of the translational inhibitor 4E-BP1, and has previously been shown to influence the translation of cyclin D mRNA^17,18^. Therefore, further analysis of protein translation focused on signal transduction through AKT and mTOR. As shown in Fig. 7a, knockdown of TRPV4 induced dephosphorylation of AKT, mTOR, p70S6K, and 4E-BP1. These findings indicated that AKT-mTOR activation was impaired in TRPV4-depleted cells. Moreover, silencing of 4E-BP1 abrogated the inhibitory effect on cyclin D3 expression and partially attenuated the growth inhibition induced by depletion of TRPV4 (Fig. 7c, d).

The products of TSC1 and TSC2 form a complex that acts downstream of AKT and upstream of mTOR to control S6K and 4E-BP1 activities in mammalian cells^19^.Therefore, the effects of TSC1 and TSC2 siRNA on cell growth in TRPV4-depleted cells were analyzed. As shown in Fig. 7e, TCS1 or TSC2 siRNA could block mTOR, p70S6K, S6 and 4E-BP1 dephosphorylation and significantly reduced the expression of cleaved PARP and Caspase3 in TRPV4 knockdown cells, suggesting that the mTOR pathway is responsible for TRPV4 knockdown-induced growth inhibition. In line with these findings, we have demonstrated that disruption of the mTOR pathway by knockdown of TSC1 or TSC2 increased cell viability and clonogenicity in TRPV4-silenced HCT-116 cells (Fig. 7f, g). Together, these results indicated that the decreased cell growth induced by TRPV4 silencing may be attributed to inactivation of the ATK-mTOR pathway in colon cancer.

### PTEN is involved in TRPV4 inhibition induced growth suppression in colon cancer cells

**Fig. 8:**
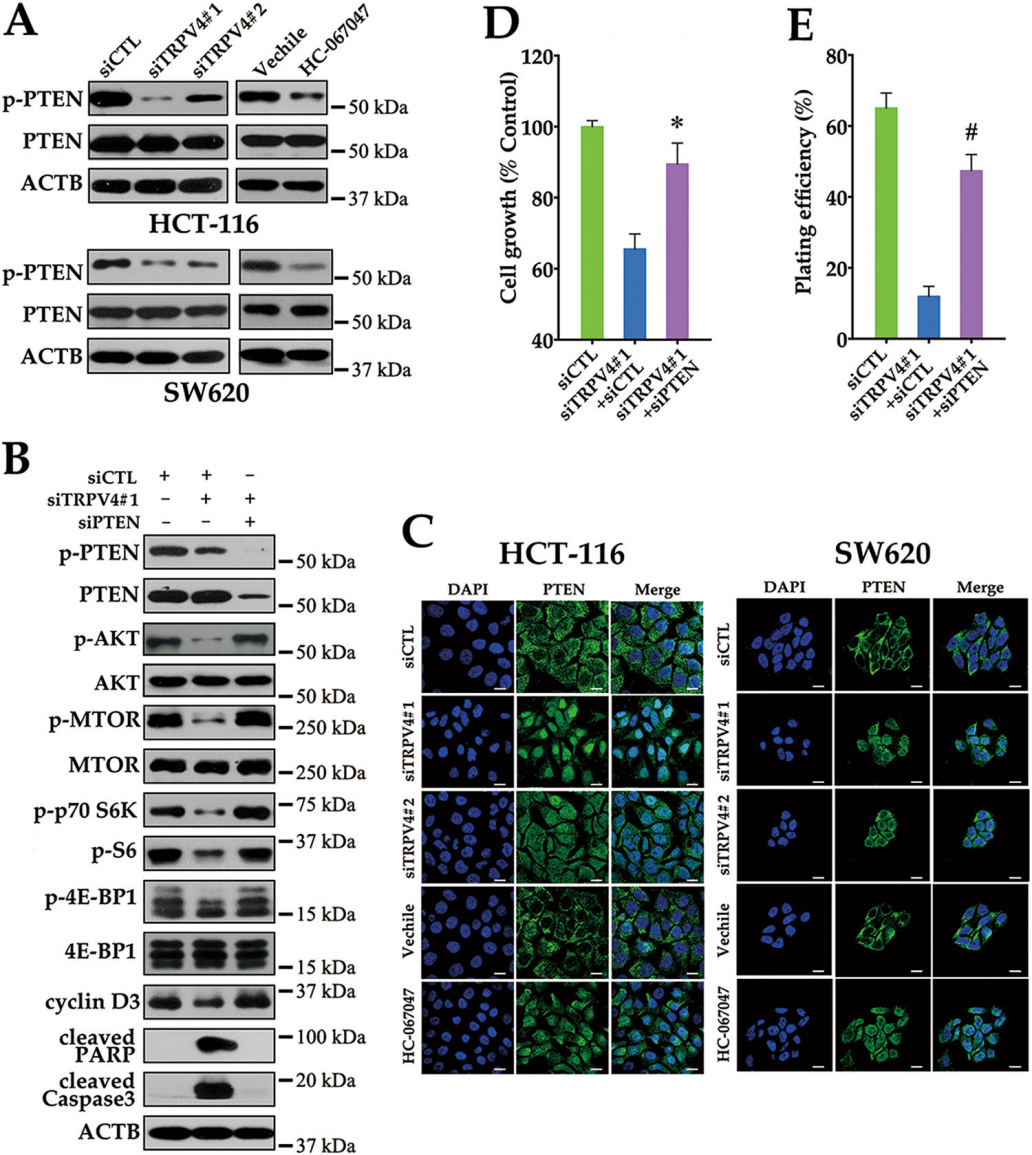
Activation of PTEN is required for the TRPV4 inhibition induced growth suppression in colon cancer. **a** Silencing of TRPV4 or HC-067047 induces dephosphorylation of PTEN. HCT-116 or SW620 cells were transfected with control siRNA (siCTL), TRPV4 siRNA#1 (siTRPV4#1) or TRPV4 siRNA#2 (siTRPV4#2) for 72 h, or treated with vehicle (0.1% DMSO) or HC-067047 (4 μΜ) for 72 h. The protein levels of phosphor-PTEN (Ser380/Thr382/383; p-PTEN), PTEN, and ACTB were analyzed by western bolt. **b** The effect of PTEN siRNA (siPTEN) on the inhibition of AKT-mTOR signaling, the decrease of cyclin D3 expression or the increase of apoptosis marker cleaved PARP and Caspase3 expression induced by TRPV4 silencing. HCT-116 cells were transfected with siCTL, siTRPV4#1 plus siCTL, siTRPV4#1 plus siPTEN for 72 h. **c** Silencing of TRPV4 or HC-067047 induces the nuclear localization of PTEN. HCT-116 or SW620 cells were transfected or treated as in (**a**). The immunofluorescent images were taken on a confocal microscope. Scale bar: 10 μm. **d** The effect of PTEN siRNA on the decrease of cell viability induced by TRPV4 silencing. Cell viability was assessed by MTT assay. **e** The effect of PTEN siRNA on the decrease of colony formation induced by TRPV4 silencing. All quantitative data shown represent the means ± SEM of at least three independent experiments. ^*^P < 0.05 and ^#^*P* < 0.001, versus the siTRPV4#1 plus siCTL group

PTEN, a dual-phosphatase that negatively regulates AKT activity, is a common tumor suppressor in human cancer^20^. We therefore asked whether activation of PTEN played a role in TRPV4-mediated AKT-mTOR dephosphorylation. Silencing of TRPV4 decreased PTEN phosphorylation, which contributed to the activation of PTEN. Similar results were obtained using the TRPV4 inhibitor HC-067047 (Fig. 8a). To further verify whether TRPV4-regulated AKT-mTOR signaling in a PTEN-dependent manner, PTEN siRNA was utilized in TRPV4-silenced colon cancer cells. As shown in Fig. 8b, PTEN siRNA attenuated the dephosphorylation of AKT, mTOR, p70S6K, S6 and 4E-BP1 in TRPV4-depleted cells. Therefore, inhibition of TRPV4 expression or activity resulted in an increase of PTEN phosphatase activity, which accounted for inactivation of the AKT-mTOR pathway. PTEN is primarily localized in the cytoplasm and opposes the function of the PI3K/AKT pathway. However, PTEN also possesses phosphatase-independent roles in the nucleus^21,22^. Interestingly, we found that TRPV4 knockdown induced nuclear localization of PTEN (Fig. 8c). Furthermore, silencing of PTEN attenuated growth inhibition and recovered the capability of clonogenicity in TRPV4 knockdown cells (Fig. 8d, e). Consistent with these findings, blocking PTEN also reduced the expression of cleaved PARP and Caspase3 in TRPV4-depleted cells. Taken together, these data indicated that PTEN participated in TRPV4-induced effects in colon cancer cell growth both through phosphatase-dependent and independent mechanisms.

## Discussion

In the present study, we reported three major findings that allow a better understanding of the role of TRPV4 in colon cancer cells. (1) We have demonstrated that TRPV4 is upregulated in colon cancer samples with poor prognosis. (2) Our biological assays in vitro and in vivo highlighted that silencing or pharmacological inhibition of TRPV4 attenuated colon cancer cell growth. (3) We demonstrated that PTEN pathway contributes to TRPV4-mediated cell growth. These clinical and biological findings have indicated the potential role of TRPV4 as a proto-oncogene in colon cancer.

Alterations in the expression of certain TRP channels are a characteristic of several types of cancer^23^. In this study, we demonstrated that TRPV4 was upregualted in human colon cancer with poor outcome. Consistent with the notion, the enhanced expression of TRPV4 is highly associated with the histological grade in human hepatocellular carcinoma^24^. However, the expression pattern of TRPV4 in colon and liver cancer is different from that in nonmelanoma skin cancer^10^. It seems that TRPV4 exhibits different expression patterns in a cancer type-dependent manner. It has previously been reported that TRPV4 channels were involved in cell proliferation, including cystic cholangiocytes^25^, sebocytes^26^, stem cells of the hippocampal dentate gyrus^27^, and tumor endothelial cells^28,29^. Although limited studies have shown that TRPV4 participated in cell proliferation in gastric and liver cancer, it has not yet been established whether TRPV4 regulated cell cycle progression to affect cancer cell growth. Here, we demonstrated that TRPV4 affected colon cancer cell growth via regulation of the cell cycle progression from the G1 to the S phase. Ca^2+^ played a crucial role throughout the mammalian cell cycle and is especially important at G1/S and G2/M phase transitions^30^. TRPC3 or TRPC6 channel-mediated Ca^2+^ influx is critical for G2/M phase transition of human ovarian cancer^31^, glioma^32^, or esophageal cancer^33^. Consistent with this notion, we showed that inhibition of the activity or expression of TRPV4 in colon cancer cells may sufficiently disrupt Ca^2+^ homeostasis to increase the proportion of cells in the G1 phase and decrease the proportion of cells in the S phase. Cyclin D1 and D3 are essential regulators of G1/S transition in response to growth factor stimulation^34,35^. A concomitant decreased protein expression of cyclin D1 and D3 was observed in TRPV4-silenced cells. However, no effect on mRNA expression was observed. These findings indicated that TRPV4 is likely a key regulator of Ca^2+^-mediated cell cycle progression through modulating the protein expression of the master G1/S transition regulators.

Modulation of TRP channels could perturb Ca^2+^ homeostasis, resulting in subsequent cell death. In hepatocellular carcinoma cells, TRPC6 is a negative regulator of cell death induced by doxorubicin, hypoxia, and ionizing radiation^36^. In contrast to TRPC6, TRPV4 is positively regulated pronounced cell death through apoptosis, oncosis, or necrosis in breast cancer or melanoma cells^11,37^. In addition, sustained exposure to TRPV4 agonists has been shown to evoke dose-dependent apoptosis of retinal ganglion cells and hippocampal neuronal cells^38^. However, we found that TRPV4 silencing by siRNA enhanced apoptosis in human colon cancer cells and decreased resistance to chemotherapy-induced apoptosis. On the other hand, TRPV4 antagonists induced apoptosis in human hepatocellular carcinoma^24^. Therefore, TRPV4 could perform two apparently opposite functions by either promoting or inhibiting apoptosis in a cell type-dependent manner. Autophagy is a self-degradative process which is associated with either cell survival or cell death^39^. Significant evidence has emerged that the functional regulation of TRP channels affected the autophagic process^40^. TRPM3 is necessary for oncogenic autophagy under starvation conditions in clear cell renal cell carcinoma^41^. TRPM2-induced Ca^2+^ influx inhibited autophagy in response to oxidative stress, causing the cells to become more susceptible to damage^42^. TRPV4 inhibited apoptosis through induction of autophagy in response to TGF-β1 stimulation in rat hepatic stellate cells^43^. In this study, we observed that TRPV4 played a role in the induction of autophagic process. Depending on the cellular context and signals, autophagy has dual functions as it has been involved in stimulating either cell survival or inducing cell death^44^. In our study, disruption of TRPV4 silencing-mediated autophagy by knockdown autophagy-related genes increased colon cancer cell viability. These results indicated that autophagy induced by TRPV4 silencing acted as a cell death mechanism.

The AKT signaling pathway regulates several normal cellular functions that are also critical for tumorigenesis. Hyperactivation of AKT is associated with increased cell growth, proliferation, cellular energy metabolism, and resistance to apoptosis^45^. In previous reports, AKT is involved in TRPV4-mediated signaling in polycystic kidneys of rats^25^ and in hippocampal neuronal cells^46^. However, the underlying mechanism of TRPV4-regulated cell growth is not completely understood. We found that the blockade of TRPV4 decreased protein levels of cyclin D1 and cyclin D3, which were regulated by translation in the mTOR signaling pathway. This suggested that TRPV4 might be involved in regulation of the mTOR signaling pathway. mTOR is a critical downstream effector of AKT, which regulates many fundamental cell processes from protein synthesis to autophagy^47^. mTOR largely regulates protein synthesis through phosphorylation of two key effectors, S6K and 4E-BP^48^. In this study, we showed that TRPV4 knockdown impaired the activation of AKT in colon cancer cells, consequently leading to inactivation of the mTOR and S6K pathway, and attenuated phosphorylation of 4E-BP1 and S6 ribosomal protein. It has been well established that mTOR controls cell cycle transition from the G1 to the S phase^18,49^. Moreover, G1 cyclins are regulated by mTOR, SK6 as well as 4E-BP1-mediated mRNA translation^17^. Collectively, TRPV4 knockdown-induced cell cycle arrest is attributed to inactivation of the AKT-mTOR pathway-mediated translation inhibition of D-type cyclins. Concomitant with the regulation of cell proliferation, mTOR, as a master regulator of cellular metabolism, also plays a crucial role in regulating autophagy^50^. In our study, inactivation of the AKT-mTOR pathway may be involved in the induction of autophagy in TRPV-depleted colon cancer cells.

Our findings that silencing of TRPV4 suppressed the AKT-mTOR pathway prompted us to investigate whether PTEN, a highly effective tumor suppressor, through negative regulation of the PI3K/AKT/mTOR pathway^51^, is involved in this process. In this study, the level of phosphorylated PTEN at Ser380/Thr382/Thr383 was significantly decreased following inhibition of TRPV4 expression or activity. These findings revealed that activation of the catalytic activity of PTEN, is in keeping with the inactivation of its downstream target AKT as well as mTOR signaling pathway. Therefore, we hypothesize that in colon cancer, abnormal expression of TRPV4 disrupted the negative regulation of AKT-mTOR signaling via sustained PTEN phosphorylation during tumor development. PTEN is primarily localized within the cytoplasm and antagonizes the function of the PI3K/AKT pathway. However it also plays important roles in chromosome stability and DNA repair and has phosphatase-independent activities in the nucleus^21,22^. In addition, the phosphorylation of PTEN at Ser380/Thr382/Thr383 can promote its nuclear accumulation^52,53^. In this study, in addition to inducing the dephosphorylation of PTEN, inhibition of TRPV4 expression or activity increased the nuclear localization of PTEN in colon cancer. In previous studies, it has been reported that cellular Ca^2+^ levels regulated the nuclear localization of PTEN through conformational interconversion with the major vault protein^54^. However, the underlying mechanisms of PTEN nuclear localization as well as its function in TRPV4-depleted cells are not well understood, and needs to be further investigated.

In conclusion, in this study we highlighted the functional importance of TRPV4 in mediating colon cancer development. Inhibition of TRPV4 suppressed colon cancer cell growth through arresting the cell cycle in the G1 phase and by inducing apoptotic as well as autophagic cell death. In addition, we provided evidence that the growth-inhibitory effect of TRPV4 knockdown is related to impaired AKT-mTOR signaling through activation of PTEN. The notion of employing the downregulation of TRPV4 activity or expression as an approach to treat human colon cancer is worthy of further investigations.

## Materials and methods

### Cell culture

The human colon cancer HT-29, HCT-116, DLD1, LoVo, Caco-2, SW480, SW620 cells were purchased from American Type Culture Collection. Cell lines were maintained in McCoy’s 5A, RPMI 1640, Ham’s F-12K, DMEM or Leibovitz’s L-15 medium supplemented with 10% fetal bovine serum, 100 U/ml penicillin, and 100 μg/ml streptomycin. All experiments were carried out in cells between passages 10 and 20. Cells were cultured at 37 °C, in 95% O_2_ and 5% CO_2_ in a humidified incubator.

### Tissue samples

Colon cancer tissue microarrays were obtained from US Biomax (Rockville, MD). The primary tumor and adjacent nontumor tissues from patients with colon cancer (*n* = 18) were obtained during operation prior to any therapeutic intervention at the Prince of Wales Hospital in Hong Kong with the approval of the Clinical Research Ethics Committee, Chinese University of Hong Kong. All subjects provided informed consent for obtaining the study specimens. The investigation conforms with the principles outlined in the Declaration of Helsinki for the use of human tissue or subjects.

### Immunohistology

IHC was performed as previously described^55^. The German semi-quantitative scoring system (no staining = 0, weak staining = 1, moderate staining = 2, strong staining = 3) and the percentages of stained cells (0% = 0, 1–24% = 1, 25–49% = 2, 50–74% = 3, 75–100% = 4) were used to assess the results. The final immune reactive score was determined by multiplying the intensity score by the percentage score, ranging from 0 to 12.

### Small interfering RNA (siRNA) transfection

ON-TARGET plus SMART pool against human TRPV4 siRNA (siTRPV4 #2) (L004195-00-0005) was obtained from Dharmacon, Inc. Other siRNAs were purchased from Genepharm. In brief, cells were transfected with 90 nM of each siRNA duplex using DharmaFECT transfection reagent according to the manufacturer’s protocol^39^. The target sequences are as following: TRPV4 (siTRPV4 #1): 5′-AUCUUGGUAACAAACUUGG-3′. ATG5: 5′-GGCAUUAUCCAAUUGGUUU-3′ and 5′-UGACAGAUUUGACCAGUUU-3′. BECN1: 5′-GAUACCGACUUGUUCCUUA-3′ and 5′ -CUAAGGAGCUGCCGUUAUA-3′. ATG7: 5′-CCAACACACUCGAGUCUUU-3′ and 5′ -GCCCACAGAUGGAGUAGCA-3′. 4E-BP1: 5′-GCAAUAGCCCAGAAGAUAA-3′ and 5′-GAGAUGGACAUUUAAAGCA-3′. TSC1: 5′-GAAGAUGGCUAUUCUGUGU-3′ and 5′-CGACACGGCUGAUAACUGA-3′. TSC2: 5′-GCAUUAAUCUCUUACCAUA-3′ and 5′-GGAGACACAUCACGUACUU-3′. PTEN: 5′-GUGAAGAUCUUGACCAAUG-3′ and 5′-GGCGCUAUGUGUAUUAUUA-3′.

### ANXA5 (annexin V) and propidium iodide (PI) staining

The cells were washed with PBS, then incubated in the binding buffer (10 mM HEPES, 140 mM NaCl, 2.5 mM CaCl_2_, 0.1% BSA, pH 7.4) containing ANXA5-FITC for 15 min. Cells were immediately exposed to 2 μg/ml PI (Sigma, P4170) before the analysis on a FACScan flow cytometer (Becton Dickinson, San Jose, CA).

### RNA isolation and reverse transcription-polymerase chain reaction (RT-PCR)

Total RNA was isolated by Trizol (Life Technologies; 15596018). RT-PCR (reverse transcription-polymerase chain reaction) was performed with the PrimeScript™ RT reagent kit (Takara; RR037A) according to the manufacturer’s instructions. To detect the mRNA levels of TRPV4, CCDN1, and CCDN3, primers used were as follows: TRPV4 forward:5′- TCACTCTCACCGCCTACTACCA-3′; reverse: 5′-CCCAGTGAAGAGCGTAATGACC-3′; CCND1 forward: 5′-TCTACACCGACAACTCCATCCG-3′, reverse: 5′-TCTGGCATTTTGGAGAGGAAGTG-3′, CCND3 forward: 5′-AGATCAAGCCGCACATGCGGAA-3′; reverse: 5′-ACGCAAGACAGGTAGCGATCCA-3′; ACTB forward: 5′-ATGTACGTTGCTATCCAGGC-3′; reverse: 5′-CTCCTTAATGTCACGCACGAT-3′. ACTB was used as internal control.

### Immunofluorescence

Cells were grown on slides and transfected with siRNAs. After 48 h transfection, cells were washed three times with PBS, fixed with 4% formaldehyde in PBS for 10 min, permeabilized with 0.1% Triton X-100 in PBS for 10 min, and then blocked with 0.5% BSA in PBS for 15 min. Slides were incubated with primary antibodies at 4 °C overnight, followed by incubation with Alexa Fluor 488-conjugated secondary antibodies for 1 h at room temperature. All antibodies were diluted with 0.5% BSA in PBS. Slides were mounted with Vectashield mounting medium and images were taken with an Olympus FV1000 confocal microscope (Olympus, Center Valley, PA) using a 60 × 1.35 NA oil objective.

### Western blot analysis

Proteins were extracted from cultured cells using a lysis buffer [20 mM TRIS-HCl, pH 7.4, 150 mM NaCl, 1 mM EDTA, 1% Triton X-100, 2.5 mM sodium pyrophosphate, 1 mM DTT, 1 mM sodium orthovanadate (Sigma, S6508), 1 μg/ml leupeptin (Sigma, L2884), 1 mM phenylmethylsulfonyl fluoride (Sigma, P7626)], followed by gentle shaking in 100–200 μl ice-cold freshly prepared lysis buffer per well for 60 min on ice. The lysate was centrifuged at 12,000 rpm for 20 min at 4 °C. The supernatant was collected and the protein concentration was determined. For western blotting, protein samples were calibrated to equal amounts, boiled with SDS-PAGE loading dye and loaded at ~ 20 μg (concentration is 2 μg/μl) into each lane of polyacrylamide gel and separated by a SDS-PAGE gel. Proteins were transferred to a nitrocellulose membrane, and the membrane was then immersed in a blocking solution containing 5% BSA and 0.1% Tween-20 in PBS for 1 h at room temperature with constant shaking. Proteins were blotted with the primary antibodies at 4 °C overnight with constant shaking. Then washed three times for 15 min each in TBST at room temperature, immunodetection was accomplished with incubation of secondary antibody conjugated with horseradish peroxidase at room temperature for 1 h, followed by reaction with chemiluminescence HRP substrate (WBKLS0500, Millipore) and fluorescent exposure to X-ray file. The relative band intensity was quantified using the AlphaEaseFC software version 6.0.0.

### Ca^2+^ imaging

A total of 5 × 10^4^ cells were seeded on coverslips and incubated with Fura-2 AM at 37 °C for 30 min in dark. Cells were washed three times with normal physiological saline solution (NPSS, 140 mmol/L NaCl, 5 mmol/L KCl, 1 mmol/L CaCl_2_, 1 mmol/L MgCl_2_, 10 mmol/L glucose, and 5 mmol/L HEPES, pH 7.4). Fura-2 fluorescence was measured using dual excitation at 340 and 380 nm using an Olympus fluorescence imaging system. Changes in [Ca^2+^]_i_ were calculated as the change in the Fura-2 ratio.

### Cell growth assay

For cell viability assay, the cells were seeded in 96 well plates at a density of 3000 cells/well overnight, transfected with siRNAs or treated with respective agents for the indicated duration and then exposed to 0.5 mg/ml MTT for 3 h at 37 °C. The formazan crystals were dissolved in DMSO. Absorbance was measured at 550 nm on Multiskan Go Microplate Spectrophotometer (Thermo, Waltham, USA) with a reference wavelength of 690 nm. For clonogenic assay, the cells were seeded into six-well plates at the density of 100 cells per well (HCT-116, HT-29, DLD1, LoVo and Caco2) or 500 cells per well (SW620), followed by incubation at 37 °C for 12d (HCT-116, HT-29 and DLD-1) or 14d (Caco-2, LoVo and SW620). Colonies were fixed with glutaraldehyde (6.0% v/v), stained with crystal violet (0.5% w/v) and imaged. Colonies with 50 or more cells were counted.

### Cell cycle analysis

Cells were fixed in ice-cold 70% ethanol at −20 °C for more than 12 h, washed twice with PBS and then exposed to PI/RNase A solution for 10 min. The cell cycle distribution was analyzed on a FACScan flow cytometer (Becton Dickinson, San Jose, CA).

### Xenograft tumorigenesis in nude mice

All animal procedures were approved by the Animal Experimentation Ethics Committee of The Chinese University of Hong Kong. HCT-116 cells (5 × 10^6^ per mouse) or SW620 cells (2 × 10^6^ per mouse) stably infected with Scramble shRNA or TRPV4 shRNA plasmid were suspended in 100 μl medium (without antibiotics), and then injected subcutaneously into the dorsal flank of the athymic nude mice (6-week-old male) (*n* = 6). The length (L) and width (W) of xenografted tumors were measured every 2 (HCT-116) or 3 (SW620) days. For the HC-067047 treatment experiments, athymic nude mice were treated with vehicle (0.1% DMSO) or HC-067047 (4 μΜ) every 2 days. Tumor volume was calculated as follows: L × (W)^2^/2.

### Statistical analysis

Statistical analysis was performed using two-tailed Student’s *t* test for comparison of two groups or one-way analysis of variance for comparison of more than two groups, followed by Tukey’s multiple comparison tests. For multiple testing, the *P* values were determined using a two-way analysis of variance with Bonferroni post-tests. All statistical analyses were carried out with the GraphPad Prism software version 5.01 (GraphPad, San Diego, CA). Data were expressed as mean ± standard error of the mean (SEM) of at least three independent experiments. A *P* value < 0.05 was considered statistically significant.
